# Ultra-Low Atomic Diffusion Barrier on Two-Dimensional Materials: The Case of Pt on Epitaxial Graphene

**DOI:** 10.1021/acsnano.5c13305

**Published:** 2025-10-01

**Authors:** Andrea Berti, Ramón M. Bergua, Jose M. Mercero, Deborah Perco, Paolo Lacovig, Silvano Lizzit, Elisa Jimenez-Izal, Alessandro Baraldi

**Affiliations:** † Department of Physics, 9315 University of Trieste, Via Valerio 2, 34127 Trieste, Italy; ‡ Polimero eta Material Aurreratuak: Fisika, Kimika eta Teknologia Saila, Kimika Fakultatea, 226245 Euskal Herriko Unibertsitatea (UPV/EHU) & Donostia International Physics Center (DIPC), M. de Lardizabal Pasealekua 3, 20018 Donostia, Euskadi, Spain; § Elettra - Sincrotrone Trieste, AREA Science Park, S.S. Km 163.5 Basovizza, 34149 Trieste, Italy

**Keywords:** surface atomic diffusion, diffusion energy, X-ray photoelectron spectroscopy, platinum, density functional theory, nucleation, graphene

## Abstract

Understanding the energetics of atomic diffusion on graphene and two-dimensional (2D) materials is critical for advancing ultraminiaturized nanodevices, where even single-atom dynamics can significantly impact their functionality and performance, and for designing next-generation catalysts with superior activity and selectivity. In this work, we demonstrate that the combination of fast, high-resolution X-ray photoelectron spectroscopy (HR-XPS) and density functional theory (DFT) simulations provides a powerful approach to probe Pt atoms diffusion on epitaxial graphene. HR-XPS with its high chemical sensitivity and temporal resolution allows *in situ* tracking of Pt 4f_7/2_ spectral components associated with monomers, dimers, and larger clusters at low temperature. This capability enabled us to monitor the rapid decay of monomer coverage and the subsequent aggregation into larger clusters. By fitting the time evolution of the different Pt species using a kinetic model, we extracted a diffusion barrier of 128 ± 6 meV, in excellent agreement with the 130 meV value obtained by nudged elastic band (NEB) calculations. These findings establish fast HR-XPS as a noninvasive, high surface-sensitive, and chemically specific technique for quantifying ultralow diffusion barriers of atoms on weakly interacting two-dimensional materials. This approach provides a practical framework for exploring surface dynamics and for guiding the controlled assembly of small atomic clusters or ordered superlattices on 2D templates.

## Graphical abstract

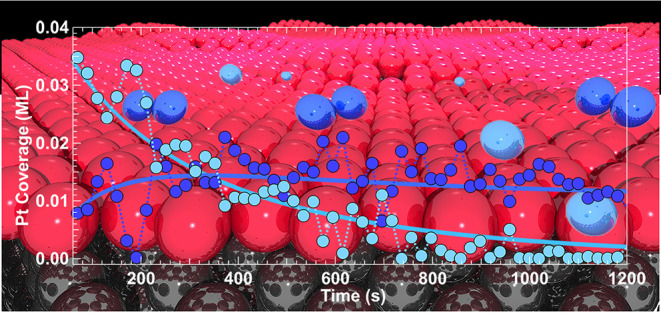

The diffusion of atoms and molecules on solid surfaces has long been a subject of large interest because of its pivotal role in catalysis, thin-film growth, electrochemistry, surface reconstruction, phase transitions and energy storage applications.^1–6^ Although extensive research over the years has been performed into the mechanisms and kinetics of atomic diffusion, the experimental observation of the dynamics of these processes at the atomic scale is still challenging. Moreover, the continuous emergence of advanced nanomaterials incorporating isolated atomic species with controlled structures has renewed and intensified the need to explore these processes further.^7–11^ As electronic and mechanical devices continue to shrink, even slight atomic-scale surface modifications compared to the ideal or predicted atomic configurations can significantly impact material performance and functionality. Consequently, understanding the energetics of atoms diffusing on solid surfaces, interfaces, and 2D materials, such as in the case of alloying and chemical reaction processes, and even in nanocrystal formation,^12,13^ remains crucial for the development of next-generation nanostructured materials. A broad array of experimental methods have been applied to investigate the atomic surface diffusion mechanisms, each offering unique advantages and facing specific challenges. Traditional microscopy-based experimental techniques, such as field ion microscopy (FIM),^14–16^ scanning tunneling microscopy (STM),^17–20^ and transmission electron microscopy (TEM)^21–23^ have long provided atomic resolution imaging capabilities to probe the dynamics of adatoms on a variety of solid surfaces. Complementary to these real-space approaches, scattering techniques such as quantum helium atom scattering (QHAS) have proven to be powerful tools for investigating ultrafast diffusion processes and ultra-low energy barriers (<50 meV).^24,25^ Photoemission electron microscopy (PEEM) has also been employed to probe atomic diffusion energetics, leveraging its chemical sensitivity and minimal perturbation of the system.^26,27^ Despite these impressive achievements, each technique exhibits inherent limitations when applied to the measurement of atomic-scale processes. Real-space imaging methods such as FIM, TEM, and STM provide atomic resolution, but generally lack chemical sensitivity, a feature that is crucial in applications requiring precise chemical specificity, for example in the formation of surface alloys.^28–30^ Moreover, these techniques can be influenced by probe-induced perturbations (tip- and high energy beam-induced effects), an issue that becomes particularly problematic when diffusion is governed by very low energy barriers.^31–33^ In contrast, QHAS is a scattering technique that effectively captures ultrafast diffusion events, although it requires a stable dynamic equilibrium and does not provide direct chemical information.^34^ Lastly, the limited spatial resolution of PEEM prevents *real-time* monitoring of individual adatoms or small clusters, often necessitating the indirect inference of diffusion barriers from the growth behavior of larger islands via application of nucleation theory rather than tracking individual atoms.^35^

The heightened interest in the functioning of atomic diffusion on solid surfaces is particularly evident in the context of metal-based single-atom catalysts (SACs). Recent research has increasingly focused on SACs, where isolated metal atoms dispersed on various carbon-based supports exhibit outstanding catalytic activity and selectivity.^36–38^ However, the practical deployment of SACs is often challenged by sintering phenomena driven by atomic diffusion, which reduce the number of active sites and alter catalyst performance under reaction conditions.^39^ Understanding the mechanisms and kinetics of atom migration and cluster nucleation is thus crucial to guiding the design of stable nanoscale architectures. Among these systems, platinum (Pt) stands out as a key element in high-performance heterostructure devices, widely used in various applications such as automotive catalytic converters, gas sensors, or fuel cells.^40–42^ Surface-deposited Pt nanostructures (single atoms, nanoclusters, and even small nanoparticles) combine high efficiency and minimal metal dosage, although they are prone to sinter, resulting in loss of active surface area. For example, recent investigations using nuclear magnetic resonance (NMR) spectroscopy to probe isolated Pt atoms on nitrogen-doped carbon supports^43^ have highlighted the growing interest in platinum-based nanostructured systems and the need for advanced spectroscopic methods capable of distinguishing chemically and structurally distinct atomic configurations often difficult to access through conventional methods. While in SACs the main challenge lies in preventing sintering through strong anchoring of isolated atoms, moving to weakly interacting supports such as graphene makes atomic mobility even more critical. Graphene, however, also offers several advantages, such as outstanding carrier mobility, thermal conductivity, optical, and mechanical properties.^44–47^ In these two-dimensional materials, the tendency of adatoms to diffuse and aggregate into clusters of different sizes can severely hinder the realization of stable SAC and nanostructured architectures. Given the central role of both graphene-based systems and Pt in many advanced technologies, a detailed understanding of Pt diffusion on graphene is thus essential. However, the very low diffusion barriers inherent to Pt adatoms on graphene, previously predicted by first-principles calculations to lie between 140 and 170 meV on free-standing graphene,^48–50^ pose significant challenges for conventional microscopy-based methods, underscoring the low-energy kinetics characteristic of adatom diffusion on weakly interacting supports.

To address these challenges, we employ a spectroscopic approach based on fast high-resolution X-ray photoelectron spectroscopy (HR-XPS), combined with density functional theory (DFT) and kinetic modeling, to investigate the evolution of individual Pt adatoms on epitaxial graphene. In our experiment, Pt atoms are deposited onto the graphene/Ir(111) interface at 45 K, and the evolution of the Pt 4f_7/2_ core-level spectra is monitored in *real time*. Supported by extensive DFT calculations, in which both adsorption energetics and core electron binding energies for nonequivalent Pt configurations are evaluated, we assign the observed spectral components to monomers, dimers, and larger clusters. The time evolution of the monomer population is quantitatively analyzed using a kinetic model, resulting in a diffusion barrier of 128 ± 6 meV, in excellent agreement with the theoretical value of 130 meV we obtain via nudge elastic band (NEB) calculations. Our investigation also deciphers the role played by structural defects in graphene (such as single vacancies, double vacancies, and Stone–Wales defects), which form during its growth process, in the diffusion of Pt atoms and their crucial involvement in the initial stages of Pt intercalation at the graphene-metal interface.

## Results and Discussion

Figure 1a illustrates a schematic of our experimental procedure, which involves the use of an evaporator to deposit Pt atoms, soft X-ray irradiation, and photoelectron kinetic energy distribution measurement. Very small Pt coverages (0.04 and 0.07 ML) were used on purpose to reduce the statistical likelihood of forming large clusters during the early stages of deposition. Importantly, under these conditions the use of a resistively heated filament evaporator ensures that the deposited species consist essentially of single Pt atoms, as the process favors the release of monomers over larger aggregates. The ability to monitor such small amounts of Pt adsorbates *in situ* is made possible not only by the high photon flux, but also by the possibility of tuning the photoionization cross-section which, at the photon energy we used, is about 1 order of magnitude higher than that of anode-based X-ray sources. Sample cooling to 45 K represents a further key ingredient to slow down the diffusion kinetics of Pt adatoms (Pt_1_), thus allowing us to monitor their aggregation into dimers (Pt_2_), and larger clusters (Pt_*n*_ with *n* > 2).

**Figure 1:**
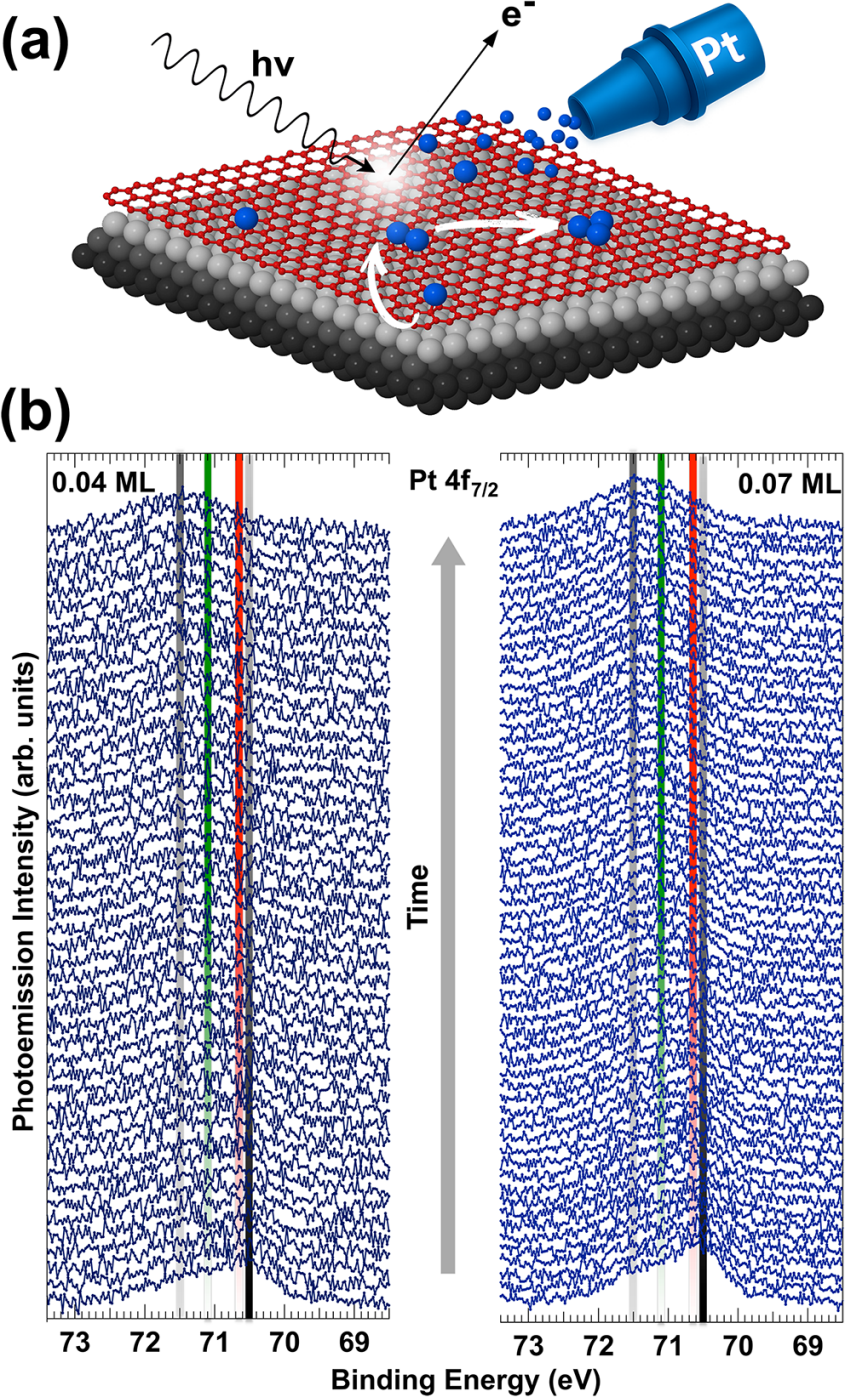
(a) Schematic representation of the experiment. Deposition of Pt atoms (blue) at 45 K on GR (red) epitaxially grown on Ir(111) (gray). White arrows suggest the atomic diffusion and nucleation processes. (b) Time-evolution of Pt 4f_7/2_ core-levels acquired (*hν* = 200 eV) in *real-time* after the deposition of 0.04 ML (left panel) and 0.07 ML (right panel) of Pt atoms. Data acquisition time was 20 s/spectrum. The first spectrum of the sequence is shown at the bottom.

The sequence of Pt 4f_7/2_ core-level spectra acquired over time after deposition and at different coverages is shown in Figure 1b. Although the variation in spectral weight during monitoring can be clearly appreciated, the ability to resolve individual spectral components is intrinsically limited by the low Pt coverages used. Nevertheless, two key features emerge: (i) a shift of the main component (70.5 eV, black line) revealed at the beginning toward higher binding energies (+0.15 eV, red line), and (ii) a progressive increase in spectral weight at even higher binding energies, with components appearing around +0.6 eV (green line) and +1.0 eV (gray line). This behavior is comparable for low and high initial coverage, as shown in the left and right panels of Figure 1b, respectively. The observed spectral changes can be consistently and qualitatively interpreted in terms of formation of Pt clusters of increasing size. According to the d-band model,^51,52^ changes in atomic coordination number in metals presenting a more than half-filled d-bands, such as Pt, lead to a progressive shift of the core-level binding energy to higher values as the coordination number increases. This trend, previously observed in surface studies,^53^ suggests that the component at the lowest binding energy can be attributed to Pt monomers, while those at progressively higher binding energies correspond to aggregates with increasing in-plane Pt–Pt coordination, since the interaction with the graphene substrate remains essentially similar across the different species.

In order to reach a quantitative understanding of the relationship between spectral evolution and Pt species, we performed in-depth DFT calculations for various Pt cluster in different configurations, ranging from monomers, which exhibit the lowest coordination, to heptamers (Pt_7_), which include the maximum coordination number achievable within the planar cluster, equal to six. Figure 2 shows the three main categories of Pt species we investigated, namely monomers, dimers, and larger clusters. The energetically favored configuration of Pt monomers is in-between two C atoms, in a bridge site, in agreement with previous determinations.^49,50^ The preferred locations is in the convex region of the corrugated Gr/Ir(111) moiré unit cell, with an adsorption energy (E_ads_) of −2.02 eV. The calculated 4f core electron binding energy for this specific configuration was taken as the zero reference of the Pt 4f_7/2_ energy scale. In the case of Pt dimers, we found two structures very close in energy. In the most stable one (*E*_ads_ = −1.38 eV) the Pt–Pt bond is normal to the surface plane, thus the dimer assumes an upright geometry with only one of the Pt atoms bonded to a carbon atom of graphene, see Figure S1 in Supporting Information. However, the corresponding 4f_7/2_ core-level shift (CLS) for the top Pt atom shows a large negative value of – 0.8 eV, which is not consistent with our experimental finding. An alternative Pt_2_ structure, only slightly higher in energy, shown in Figure 2 (Δ*E*_ads_ = +0.05 eV) features a planar configuration in which both Pt atoms lie again in equivalent bridge sites, thus forming a planar dimer with an interatomic distance of 2.60 Å. This geometry yields a CLS of about +0.15 eV for both atoms, in good agreement with the shift toward higher binding energies observed in the experiment. This apparent inconsistency between spectral behavior and computed energetics can be reconciled by calculating the diffusion barrier of Pt monomers on graphene and the formation pathways of Pt dimers. Our NEB results reveal that Pt atoms can diffuse between adjacent bridge sites on graphene with a very low energy barrier of 130 meV, which increases the likelihood of two atoms crossing paths during the diffusion process and forming a dimer. However, once a planar dimer is formed, the transition to the thermodynamically most stable configuration, namely the vertical structure, requires overcoming an energy barrier of 520 meV, which is significantly larger than the monomer diffusion barrier. Given the low thermal energy at 45 K, within the experimental time scale of our data acquisition of approximately 10^3^ seconds, the formation of vertical dimers is kinetically hindered. This finding is consistent with the formation of planar rather than three-dimensional clusters, when Pt is deposited at room temperature on the graphene/Ir interface.^54,55^ For clusters larger than dimers, specifically trimers (Pt_3_), tetramers (Pt_4_), and heptamers (Pt_7_), the most stable configurations are always three-dimensional clusters, with some atoms not bonded to the support and instead forming a second Pt layer, i.e., with a similar behavior to what was found in the case of Pt clusters on free-standing graphene.^7^ This finding can be explained considering the stronger Pt–Pt interaction with respect to the Pt–C one. Following the same rationale applied to dimers, we also searched for the most stable planar structures for larger clusters, whose structures and adsorption energies are reported in Figure 2. Interestingly, for clusters formed by more than three atoms we observe a pinning of the graphene layer as already reported in the case of Pt_12_ and Pt_13_.^56^ For these larger 2D clusters (*n* ≥ 3), DFT calculations yield a broad, overlapping range of Pt 4f_7/2_ CLSs, spanning from +0.3 to +0.7 eV, reflecting the diversity of local coordination environments. The possible presence of multiple isomeric configurations, associated to clusters with the same nuclearity but different geometries, may further contribute to the spectral broadening. Given this spread, combined with the intrinsic lifetime broadening of the Pt 4f_7/2_ core-level, individual spectral features associated with specific geometries cannot be unambiguously resolved. Therefore, the theoretically predicted range is consistent with the experimentally observed broad component centered around +0.6 eV, which we accordingly assign to the aggregation of Pt adatoms into clusters larger than dimers. However, the spectral evolution shown in Figure 1b also reveals the presence of an additional component, located at about +1.0 eV relative to Pt_1_, which cannot be accounted for by any of the calculated CLSs for 2D or 3D clusters, including those with *n* ≥ 3. This discrepancy suggests the existence of a different class of Pt species responsible for the high-binding-energy spectral region.

**Figure 2:**
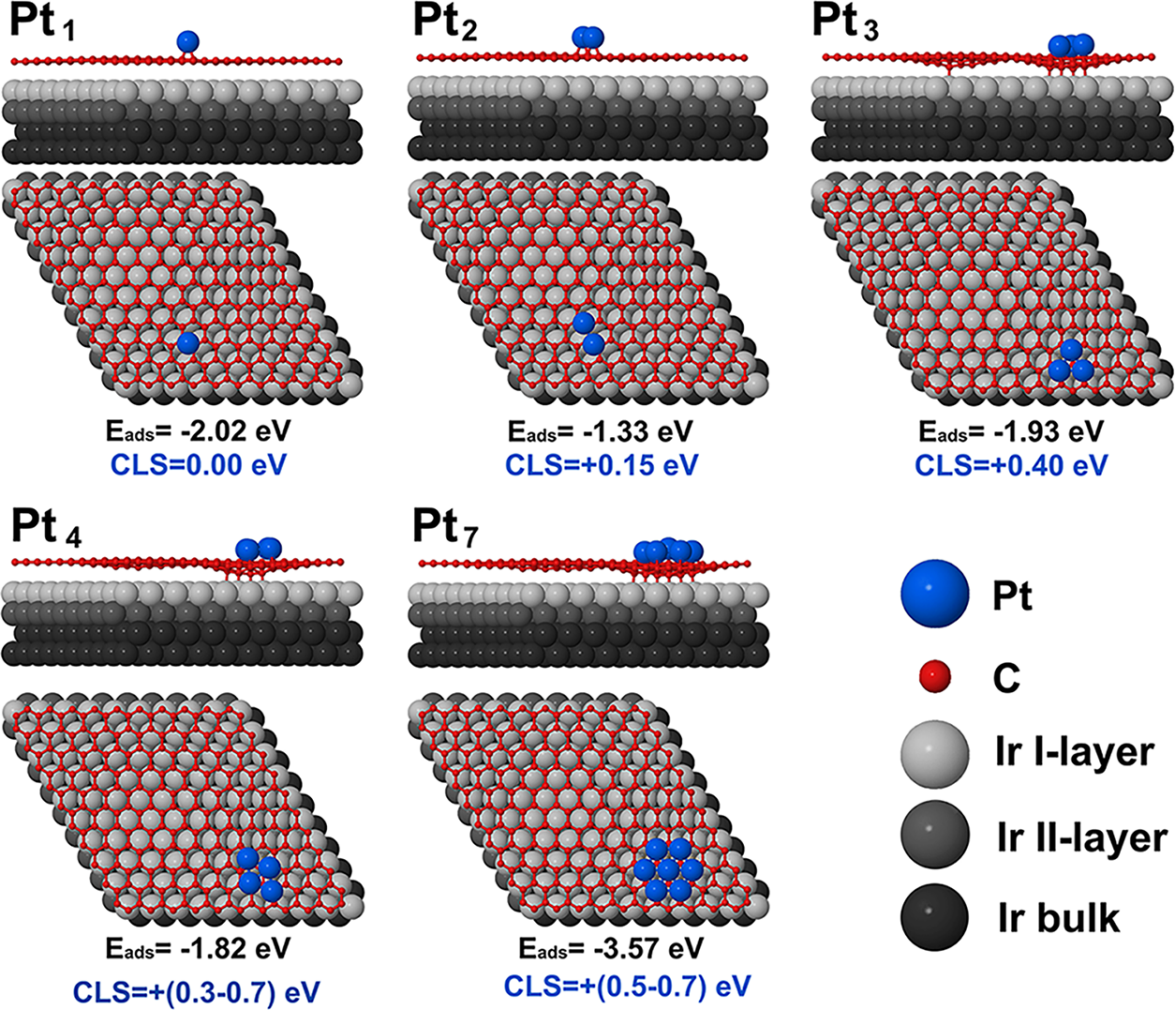
DFT calculated adsorption energies and Pt 4f core-level shifts for the investigated structural models corresponding to monomers (Pt_1_), dimers (Pt_2_), trimers (Pt_3_), tetramers (Pt_4_) and heptamers (Pt_7_). Pt atoms are depicted in blue, C in red, while Ir atoms are showing a gradient from gray to black, depending on the layer.

In order to understand the origin of the high energy spectral components we decided to investigate the role played by defects in graphene. It is known that, although graphene on Ir(111) extends over very large surface areas and even covers the individual monatomic steps, the defects that arise in the growth process cannot be neglected.^57,58^ We first focused on the most common defects observed in epitaxial graphene grown on metal substrates^57,59,60^ (see Figure S2 in the Supporting Information): single vacancy (SV), double vacancy (DV), and Stone–Wales (SW) defect. SV occur when a single carbon atom is removed from the graphene lattice, leaving behind a vacancy. DV involves the removal of two adjacent carbon atoms and can reconstruct into different configurations: (1) the triangular 555–777 form, consisting of three pentagons and three hexagons and (2) the butterfly 5555–6–7777 form, involving four pentagons, one hexagon, and four heptagons. SW defects arise from a local bond rotation that creates a pair of pentagons and heptagons within the lattice. Given the low diffusion barrier of Pt monomers, we expect these defective configurations to act as sites of adsorption for Pt adatoms and even as potential nucleation sites. The key findings from our DFT calculations are summarized below, with a detailed discussion provided in Figure S3 of the Supporting Information. For SV, the removal of one C atom generates a localized defect where the Pt adatom adopts a quasi-hollow position, coordinating with the three adjacent carbon atoms (*E*_ads_ = −6.28 eV). This configuration leads to a large CLS of +1.92 eV. In the 555–777 DV configuration, the Pt monomer preferentially adsorbs at a bridge site between two adjacent heptagon rings (*E*_ads_ = −2.99 eV), with a CLS of +0.23 eV. In the alternative 5555–6–7777 DV configuration, Pt can bind at the bridge site along the interface between two heptagons rings (*E*_ads_ = −3.45 eV), with a CLS of +0.23 eV. At SW defects, the Pt monomer also prefers a bridge site along the pentagon–heptagon interface (*E*_ads_ = −2.47 eV), resulting in a CLS of +0.26 eV. Importantly, among all configurations investigated, the theoretically computed CLS values predominantly fall either well below or significantly above the measured CLS value, which is approximately +1.0 eV. Such a discrepancy between experiment and theory suggests that these adsorption configurations do not account for the high binding energy 4f component observed in the spectra, leading us to consider alternative scenarios. We next explored the possibility of Pt diffusion through these defect sites, a mechanism previously observed in several graphene/metal interfaces, which, at high temperature and high coverage, is considered as the first step in the formation of intercalated atomic layers between graphene and the metal substrate.^8,61–63^ Building on this hypothesis, we theoretically investigated the possibility of Pt atoms passing through graphene defects and sitting between the graphene layer and the Ir substrate. For each defect type, multiple configurations were considered. In Figure 3, only the most relevant results are summarized, while a comprehensive overview of all configurations is provided in Figure S4 of Supporting Information. In the case of SV, the graphene lattice reconstructs, with the dangling carbon atom forming a bond to the Ir substrate. Upon Pt intercalation, the Pt monomer occupies a hollow site on the Ir surface, coordinated by three Ir atoms, with the adjacent carbon atoms pushed further downward. This configuration is found to be highly stable, with an adsorption energy of −8.98 eV. However, the associated CLS exceeds +3 eV, which is also inconsistent with the experimental observations. In the 555–777 DV structure, the Pt monomer intercalates between three Ir atoms directly beneath a carbon atom at the intersection of the heptagon rings. The adsorption energy ranges from −6.46 eV (at the three-heptagon intersection) to −5.77 eV (beneath the pentagon-heptagon junction), with corresponding CLS values between +1.77 and +1.17 eV. In the 5555–6–7777 structure, Pt similarly occupies hollow sites beneath the graphene layer, with adsorption energies between −6.18 and −5.64 eV, and CLS values ranging from +1.39 to +1.14 eV. Lastly, for SW defects, the Pt monomer is found intercalated among three Ir atoms and the adsorption energies vary between −6.17 eV (beneath the intersection of two heptagons) and −5.97 eV (beneath the junction of pentagon, hexagon, and heptagon), with associated CLS values ranging from +1.26 to +0.90 eV. These findings show that the calculated CLS values for Pt intercalated at DV and SW defects fall between +1.39 and +0.90 eV, in good agreement with the experimentally observed high Pt 4f core electron binding energy component. This scenario strongly supports the assignment of the last experimentally detected spectral component at approximately 1 eV to intercalated Pt atoms. Notably, our results are consistent with previous studies indicating that DV (555–777 and 5555–6–7777) and SW defects are the most common in epitaxial graphene grown on metal substrates, whereas SVs are less frequently observed.^64,65^ To further support the assignment of the high-binding-energy spectral component to intercalated Pt atoms, we conducted a detailed analysis of the Ir 4f_7/2_ core-level spectrum measured at the end of the deposition/diffusion process. The Ir(111) spectrum, both in the clean and graphene-covered surface, is known to consist of two components separated by a surface core-level shift (SCLS) of −550 meV, originated by bulk (60.87 eV) and first-layer (60.32 eV) atoms.^66^ The 4f_7/2_ spectrum measured after the diffusion process at 45 K shows a slightly modified line shape, as shown in Figure 3c. In order to obtain a good fit, a third component placed at −370 ± 50 meV with respect to the bulk one, has to be included in the data analysis. To reveal the origin of this peak we calculated for all Pt atoms in defective configuration the Pt-induced Ir SCLSs. We expect indeed that, as for the case of adsorbates^66–69^ the formation of a chemical bond between Pt and Ir atoms would induce a substantial modification in Ir core electron binding energies. In all the investigated configurations, the intercalated Pt monomer is located at a hollow site on the Ir surface, coordinated to three Ir atoms. As a representative example, Figure 3c illustrates the case of Pt intercalated beneath the intersection of one heptagon and two hexagons in a SW defect, where the computed CLS for Pt 4f_7/2_ is +1.01 eV. The three Ir atoms located directly beneath the intercalated Pt exhibit distinct 4f_7/2_ SCLS values of −350, −380, and −390 meV, in good agreement with the experimentally observed value of −370 meV. These findings further reinforce the identification of the diffusion of Pt adatoms through the defects as the origin of the high-binding-energy component in the Pt 4f_7/2_ spectra. In addition, high-resolution XPS measurements were used to estimate the order of magnitude of the defect density in the graphene layer, including single and double vacancies or Stone–Wales defects. Based on DFT calculations performed on both Highly Oriented Pyrolytic Graphite (HOPG)^70^ and graphene,^71^ we estimate a defect concentration of approximately 1%. Considering the different atomic densities of graphene and Pt, this density of defects is sufficient to accommodate the amount of intercalated Pt observed in our experiments, about 0.02 ML, thus supporting the proposed interpretation. The corresponding binding energy shift, extracted through a fit in which the intercalated Pt component was allowed to vary within the DFT-predicted CLS range, was determined to be +1.10 eV. Further details on the fitting procedure are provided in the Supporting Information (Figure S5). It is important to note that this process represents only the initial stage of intercalation, where Pt atoms become incorporated at defect sites, then Pt can further diffuse beneath the graphene layer, migrating away from the defects and leading to a more extended interfacial layer between the C network and the metal support.^8^

**Figure 3:**
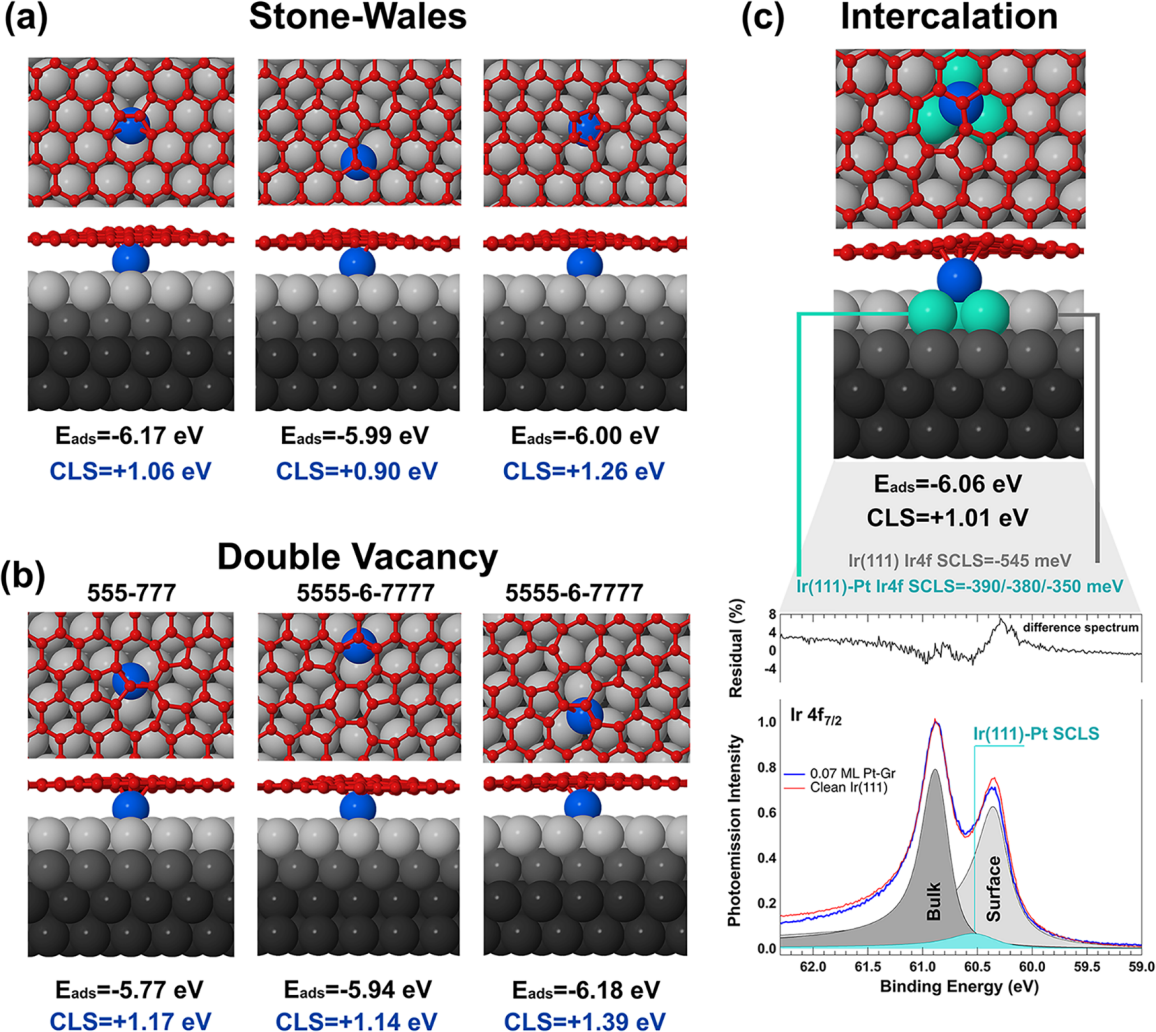
DFT calculated adsorption energies and CLSs for intercalated Pt atoms in (a) Stone–Wales defects and (b) double vacancies. (c) Calculated CLS for Ir surface atoms bonded to intercalated Pt and Ir 4f_7/2_ core-level spectra acquired after Pt deposition, with bulk (dark gray), surface (light gray), and Pt-bonded (light blue) components indicated. In the corresponding structural models, Pt atoms are shown in red, while C and Ir atoms are depicted in gray and blue, respectively.

After DFT identification of all the spectral components experimentally observed, we proceeded with the analysis of the Pt 4f_7/2_ spectral sequences through a χ^2^ minimization^72,73^ to optimize the line shape parameters for each Pt species and determine its populations. In the following, we focus on the analysis of the data set acquired after deposition of 0.07 ML of Pt, which provides a higher signal-to-noise ratio and allows a more robust spectral decomposition. The lower coverage case (0.04 ML) leads to consistent results and is discussed later. To reduce the degrees of freedom in the data analysis due to overparameterization, we assumed that monomers and dimers share the same line shape. Conversely, a different line shape was constrained to larger clusters and intercalated Pt, as these species are expected to exhibit increased inhomogeneous broadening. Additionally, we analyzed the correlation matrix of the Pt 4f_7/2_ core-level spectra to assess parameter dependencies and computed χ^2^ maps, which quantify how χ^2^ varies as a function of the fitting parameters. These maps help identify regions of parameter space that yield the best agreement with the experimental data (see Figure S5 in the Supporting Information). As expected, the Lorentzian width was found to be 0.45 eV for all species, consistent with the intrinsic lifetime broadening of Pt 4f electrons, as reported in previous studies.^53^ Meanwhile, the Gaussian width was found to be larger for intercalated Pt and larger clusters (0.61 eV ± 0.02 eV) compared to monomers and dimers (0.54 ± 0.02 eV), accounting for the presence of a variety of possible configurations. Once these parameters were determined, we proceeded to fit the entire evolution of the Pt 4f_7/2_ spectra, as shown in Figure 4, with the aim of determining the coverage evolution of the different Pt species over time. The starting time for the evolution (*t* = 0) corresponds to the end of Pt deposition, with the first spectrum acquired approximately 60 s later, the minimum time required to set the sample acquisition position and start the first scan. Subsequent spectra were collected every 20 s, with the full measurement spanning about 1200 s. The monomer population (black circles in Figure 4b) decreases rapidly from the very beginning, as adatoms diffuse and aggregate into dimers and larger clusters. As expected, the intensity of the dimer component (red dots in Figure 4b) rises quickly, reaching a maximum before stabilizing, while larger clusters continue to increase longer. The populations intersect at around 330 s after the end of deposition, marking a key transition point in the structural evolution of the system. The diffusion behavior of Pt observed experimentally and complemented theoretically in this work, reminisces metal sintering occurring via Ostwald ripening mechanism. Indeed, it was previously demonstrated that, at the subnanocluster size regime, Pt sinters through adatoms migration.^74^ To provide a quantitative interpretation of the observed trends and determine the atomic diffusion barrier for Pt adatoms, we developed a kinetic model based on a set of differential equations. Importantly, the observed diffusion process is thermally activated, with the energy required to overcome the barrier supplied by phonon-mediated lattice vibrations. Under our experimental conditions, no external perturbations are introduced during the measurements, so atomic mobility can only be tuned through the substrate temperature. One of the conditions imposed in our model is that at 45 K only monomers are mobile, enabling them to diffuse and coalesce into larger clusters. This is supported by our NEB calculations, which show that dimers have a significantly higher diffusion barrier (270 meV). To further validate this assumption, we evaluate the jump rates using an Arrhenius-like expression for the rate constants,^75,76^ given by $k_{i}=\nu_{0}e^{\left(-\frac{E_{d}}{k_{B}T}\right)}$ where ν_0_ is the pre-exponential factor, *k*_B_ is the Boltzmann constant, *T* is the temperature, and *E*_d_ is the associated energy barrier. Assuming a typical surface diffusion prefactor ν_0_ = 10^13^ Hz, the DFT calculated monomer diffusion barrier E_d_ = 130 meV yields a jump rate of approximately 2.8 × 10^–2^ s^–1^. In contrast, for dimers, the significantly higher diffusion barrier of 270 meV drastically reduces the jump rate by more than 16 orders of magnitude. Consequently, under our experimental conditions and time scale, both dimers and larger clusters can be considered effectively immobile relative to monomers. Furthermore, our model neglects the fragmentation of larger clusters, given that we calculated a dissociation energy of dimers equal to 1.21 eV. Based on these conditions, the evolution of the populations for each cluster size (up to heptamers) is governed by the following set of differential equations

1 $$\begin{array}{l} \frac{dN_{1}}{\mathrm{dt}}=-2k_{1}N_{1}^{2}-k_{2}N_{1}N_{2}-k_{3}N_{1}N_{3}-k_{4}N_{1}N_{4}-k_{5}N_{1}N_{5}-k_{6}N_{1}N_{6}\\ \frac{dN_{2}}{\mathrm{dt}}=k_{1}N_{1}^{2}-k_{2}N_{1}N_{2}\\ \frac{dN_{3}}{\mathrm{dt}}=k_{2}N_{1}N_{2}-k_{3}N_{1}N_{3}\\ \frac{dN_{4}}{\mathrm{dt}}=k_{3}N_{1}N_{3}-k_{4}N_{1}N_{4}\\ \frac{dN_{5}}{\mathrm{dt}}=k_{4}N_{1}N_{4}-k_{5}N_{1}N_{5}\\ \frac{dN_{6}}{\mathrm{dt}}=k_{5}N_{1}N_{5}-k_{6}N_{1}N_{6}\\ \frac{dN_{7}}{\mathrm{dt}}=k_{6}N_{1}N_{6} \end{array}$$

where *N*_1_, *N*_2_,..., *N*_7_ denote the populations of monomers, dimers, trimers, *etc*., up to heptamers, and *k*_1_, *k*_2_,..., *k*_6_ are the respective rate constants. Since the kinetics are dominated by monomer diffusion, owing to their very low diffusion barrier and high mobility, we assume that all *k*_*i*_ are effectively equal, following an Arrhenius-like behavior. We then used this set of differential equations to fit the experimentally determined coverage evolution over time. In particular, the solutions obtained through Euler’s method^77^ were employed in a χ^2^ minimization procedure (see Figure 4c), and are illustrated as continuous lines in [Fig fig4]b. Being the temperature of the substrate of 45 ± 2 K, the atomic diffusion barrier was determined to be 128 ± 6 meV, which is in excellent agreement with the theoretical value of 130 meV obtained using NEB. In addition, by applying the same χ^2^ minimization procedure, the pre-exponential factor was determined to be 10^13 ± 0.5^ Hz, consistent with typical values of 10^12^–10^13^ Hz reported for most surfaces.^76^ To account for the possible correlation between E_*d*_ and ν_0_, the behavior of the pre-exponential factor as a function of the activation energy was also evaluated, and found to be minor in the range of the activation energies explored. The proposed kinetic model accurately reproduces the rapid decrease in monomer coverage as Pt adatoms diffuse and aggregate. Moreover, the evolution of the dimer population is well captured, with the dimer intensity rising sharply before stabilizing with only a subtle decrease. The intersection of the different coverage trends also aligns closely with our experimental observations. On the contrary, the agreement between experiment and theory is quantitatively less accurate in the case of larger clusters, where the broad and overlapping spectral contributions (arising from several low-lying isomers identified by DFT and the simultaneous presence of intercalated Pt) complicate the deconvolution and limit a direct component-by-component comparison. This discrepancy reflects the assumption made in the spectral analysis, where each Pt species is represented by a single component. While this approximation is well justified for monomers and dimers, whose well-defined geometries lead to narrowly distributed and consistent CLS values, it becomes more stringent for larger clusters and intercalated atoms, which encompass a broader variety of local configurations and resulting shifts. Nevertheless, the agreement between theory and experiment observed for monomer and dimer populations indicates that the model captures the essential aspects of the underlying kinetics and supports the reliability of the extracted parameters. In particular, model reproduces in remarkable way both the low temperature time scale and the coverage evolution of the experimental data, especially considering that even minor variations in the diffusion barrier significantly alter the predicted monomer decay trend, as shown and further detailed in Figure S6 of the Supporting Information. Applying the same procedure for the lower coverage data set, see Figure S7 in Supporting Information, despite the added complexity of a reduced signal-to-noise ratio, yields analogous results for the Pt adatom diffusion barrier, with *E*_d_ = 126 ± 6 meV, which remains in excellent agreement with theoretical predictions, further confirming the robustness of our analysis. These results highlight the capability of our spectroscopic approach to experimentally detect the atomic diffusion barrier, even in a weakly interacting system such as Pt deposited on graphene. Beyond Pt on graphene, this methodology can readily be extended to other metals on 2D support systems, enabling real-time studies of nucleation, growth, and sintering phenomena. The key requirement is the presence of sufficiently narrow core-level features, as is the case for many 4d and 5d transition metals with 3d and 4f levels, such as Au and Ag on MoS_2_ ^78^ or Ir and Au on graphene/Ru(0001).^50^ In these systems, theoretical studies have also predicted ultralow diffusion barriers, making them attractive candidates for our approach. Similarly, light elements such as Li and Al on graphene have been predicted to combine low diffusion barriers with sufficiently narrow 1s and 2p levels,^79^ thus broadening the range of systems where this methodology can be applied. Future works could apply this spectroscopic and modeling framework to investigate even more complex architectures, such as intercalated atoms, nanoalloy kinetics,^80^ and even stable arrayed single atoms,^81^ where chemical sensitivity plays a key role. Such insights may guide the design of improved nanoscale catalysts, electronic devices, and self-assembled nanostructures.

**Figure 4:**
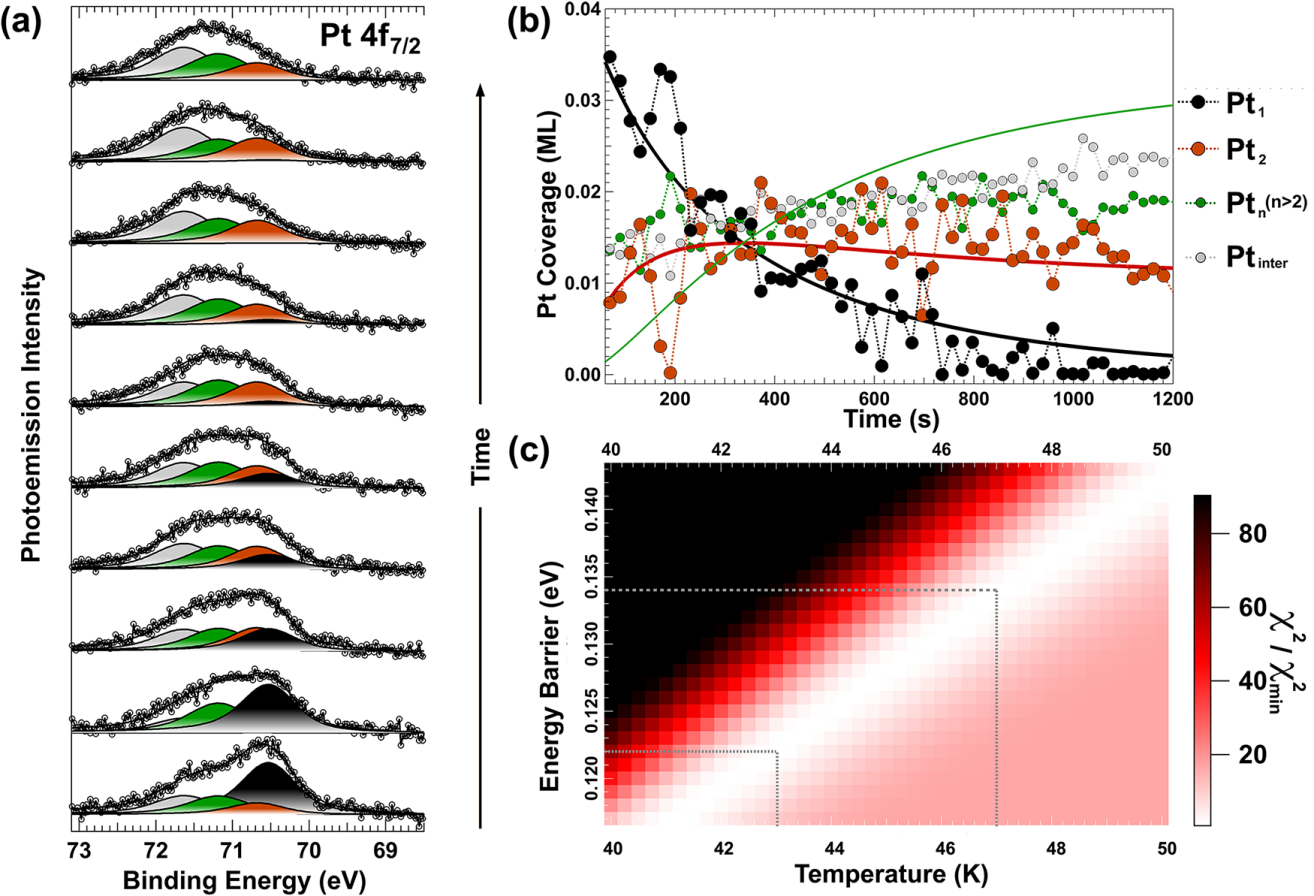
(a) Fit of the evolution of the 4f_7/2_ core-level spectra acquired in *real-time* with photons of energy *hν* = 200 eV immediately after deposition. For clarity, one out of every six spectra is shown, although all spectra were used in the analysis. The contributions from monomers (black), dimers (red), larger clusters (green), and intercalated Pt (gray) are indicated. (b) Temporal evolution of the coverages of the various platinum species on the surface. Also shown is the best-fit solution from the differential equations, corresponding to a temperature of 45.75 K and a diffusion barrier E_d_ of 130 meV. (c) Two-dimensional χ^2^ map illustrating the dependence of the fit on T and E_*d*_, obtained by systematically varying these parameters in the kinetic model.

## Conclusions

We have demonstrated that fast HR-XPS provides a direct, nonperturbative, and chemically sensitive tool for monitoring adatom diffusion on weakly interacting two-dimensional supports. By depositing Pt at 45 K on epitaxial graphene/Ir(111) and tracking *in situ* the spectral signatures of monomers, dimers, and larger clusters, we observed a rapid decay of monomer coverage followed by cluster growth via an Ostwald-ripening mechanism. The evolution of the system was captured by a kinetic model, which not only reproduces the temporal evolution of Pt populations but also yields an experimental diffusion barrier of 128 ± 6 meV, in close agreement with the value of 130 meV obtained via NEB calculations. These findings establish the combination of HR-XPS and DFT as a powerful approach for quantifying ultralow diffusion energies that govern adatom mobility.

## Methods

### Experimental Methods

All measurements were carried out at the SuperESCA beamline^82,83^ of the Elettra synchrotron radiation facility (Trieste, Italy). The ultrahigh vacuum (UHV) system (base pressure <1× 10^–10^ mbar) is equipped with a sputter ion gun for sample cleaning, a mass spectrometer, low-energy electron diffraction (LEED) optics, and a SPECS PHOIBOS 150 hemispherical electron energy analyzer with a delay line detector. The Ir(111) single crystal used for the graphene growth was first cleaned through several cycles of *Ar* ^+^ sputtering and annealing up to 1470 K, followed by exposure to oxygen (three ramps up to 1070 K with an O_2_ pressure of 5 × 10^–7^ mbar) to remove residual C atoms. The residual O was finally removed by exposing to H_2_ in a temperature range between 370 and 770 K and with a pressure of 1 × 10^–7^ mbar. A final flash up to 570 K was then sufficient to remove the small amount of residual H. The sample cleanliness and ordering were verified by HR-XPS and LEED, respectively. The measurements reported in this work were also performed in UHV conditions. The cleanliness of the Ir surface was checked by measuring the Ir 4f_7/2_ core-level, which showed the typical surface core-level shift to lower binding energies, equal to -550 meV,^66^ and by verifying the absence of contaminants such as C, O, S and B within our detection limit (about 0.1% ML). Graphene was grown using a well-established procedure.^84,85^ Initially, the Ir(111) surface was exposed to a C_2_H_4_ pressure of 5 × 10^–8^ mbar at a temperature of 470 K for 2 min. Then followed five cyclic temperature ramps between 500 and 1420 K and two final ramps with an increased 3 × 10^–7^ mbar background pressure. This procedure results in the formation of a moiré superstructure, where (10 × 10) graphene unit cells align with (9 × 9) unit cells of the Ir(111) substrate.^86^ The weak graphene-substrate interaction leads to a low-corrugation graphene monolayer.^87^ Pt atoms were deposited by means of a sublimation process from a high purity (99.995%) Pt wire resistively heated, with the sample kept at 45 K. This deposition method is known to predominantly produce single atoms, since thermal evaporation from a filament strongly favors monomer desorption over the emission of dimers or clusters.^88^ The evaporation rate was calibrated in situ via HR-XPS by comparing the Pt 4f_7/2_ signal with the Ir 4f_7/2_ signal from the surface component acquired on clean Ir(111), taking into account the tabulated photoionization cross sections. The resulting evaporation rate was 0.04 ML/min, where 1 ML corresponds to the surface density of the Ir atoms in the Ir(111) surface (1.57 × 10^15^ atoms/cm^2^). High resolution and fast XPS Pt 4f_7/2_ core-level spectra were acquired with a photon energy of 200 eV, an overall energy resolution of 50 meV, and a data acquisition time of about 20 s/spectrum. Likewise, the Ir 4f_7/2_ core-level spectra were collected using also photon energies of 200 eV. All the spectra were measured in the normal emission geometry and the core–electron binding energy (BE) is referenced to the sample Fermi level, acquired after each spectrum. The peak-fit analysis was carried out using a combination of Doniach–Šunjić (DS)^89^ profiles convoluted with a Gaussian. The line shape parameters are the Lorentzian width (Γ), the Anderson singularity index (α), and the Gaussian width (*G*), the latter accounting for phonons, experimental and inhomogeneous broadening. A linear background was subtracted from the core-level photoemission signal.

### Computational Methods

Plane-waves density functional theory (PW-DFT) calculations were carried out using the Vienna Ab initio Simulation Package (VASP),^90,91^ employing the Perdew–Burke–Ernzerhof (PBE) exchange-correlation functional^92,93^ and the Projector Augmented-Wave (PAW) method^94^ to describe the interactions between core and valence electrons. A plane-wave cutoff energy of 400 eV was applied and spin polarization was included. Partial occupancies were treated using Gaussian smearing with a width of 0.1 eV. van der Waals interactions were included using the Grimme D3 dispersion correction scheme.^95^ The Brillouin zone was sampled at the Γ point.

The low-corrugation epitaxial graphene on Ir(111) was modeled using a sufficiently large supercell that includes structural variations across the surface. Specifically, the previously constructed (9 × 9) Ir(111) slab comprising four atomic layers (lattice parameter of 2.74 Å) was modeled with a (10 × 10) graphene layer placed on top of the slab.^96^ The two bottom layers of Ir were fixed, while the upper two layers and graphene were allowed to move during relaxations. An additional 14 Å vacuum gap was added to avoid interactions between periodic images in the z direction.

To explore the energy landscape of adsorbed Pt clusters, a global minima search was performed by optimizing randomly generated structures of surface deposited Pt, ranging from monomers to heptamers, using the second order Bond Length Distribution Algorithm (S-BLDA) implemented in PGOPT by Zhai et al.^97,98^ All structures were relaxed with a convergence criterion of 10^–6^ and 10^–5^ eV for the electronic energy and the geometry relaxation, respectively. Energy barriers were obtained using the climbing-image nudged elastic band (CI-NEB) calculations.^99^ The transition states were confirmed by the presence of a unique imaginary frequency. The core-level binding energies were computed within the final-state approximation, thus including the effects of electronic core–hole screening upon excitation.

The adsorption energy of the Pt*_n_* to the support was calculated as

2 $$E_{\mathrm{ads}}=E\left(Pt_{n}/\mathrm{support}\right)-E\left(Pt_{n}/\mathrm{gas}\right)-E\left(\mathrm{support}\right)$$

## Supplementary Material



## Notes

DFT-calculated adsorption energies and core-level shifts for Pt dimers and extended set of monomers on epitaxial graphene; Structural models of graphene defects; details of the data analysis of Pt 4f_7/2_ core level spectra for the determination of line shape parameters; Experimental results at low Pt coverage with kinetic modeling and diffusion barrier extraction (PDF)
