# Supplementary material for: Ultra-Low Atomic Diffusion Barrier on Two-Dimensional Materials: The Case of Pt on Epitaxial Graphene
Source: ACS Nano. 2025 Oct 1;19(40):35921–32. doi: 10.1021/acsnano.5c13305 (PMC12530044; doi:10.1021/acsnano.5c13305)
Supplement: Supplementary file 1 [file nn5c13305_si_001.pdf]

Supporting Information
Ultra-Low Atomic Diffusion Barrier on Two-Dimensional Materials: The Case of Pt on Epitaxial Graphene

Andrea Berti,[†] Ramón M. Bergua,[‡] Jose M. Mercero,[‡] Deborah Perco,[†] Paolo Lacovig,[¶] Silvano Lizzit,[¶] Elisa Jimenez-Izal,[‡] and Alessandro Baraldi[*,†,¶]

†*Department of Physics, University of Trieste, Via Valerio 2, 34127 Trieste, Italy*

‡*Polimero eta Material Aurreratuak: Fisika, Kimika eta Teknologia Saila, Kimika Fakultatea, Euskal Herriko Unibertsitatea (UPV/EHU) & Donostia International Physics Center (DIPC), M. de Lardizabal Pasealekua 3, 20018 Donostia, Euskadi, Spain*

¶*Elettra - Sincrotrone Trieste, AREA Science Park, S.S. Km 163.5 Basovizza, 34149 Trieste, Italy*

E-mail: alessandro.baraldi@elettra.eu

Pt Dimer in Upright Adsorption Geometry: Global Energy Minimum Configuration

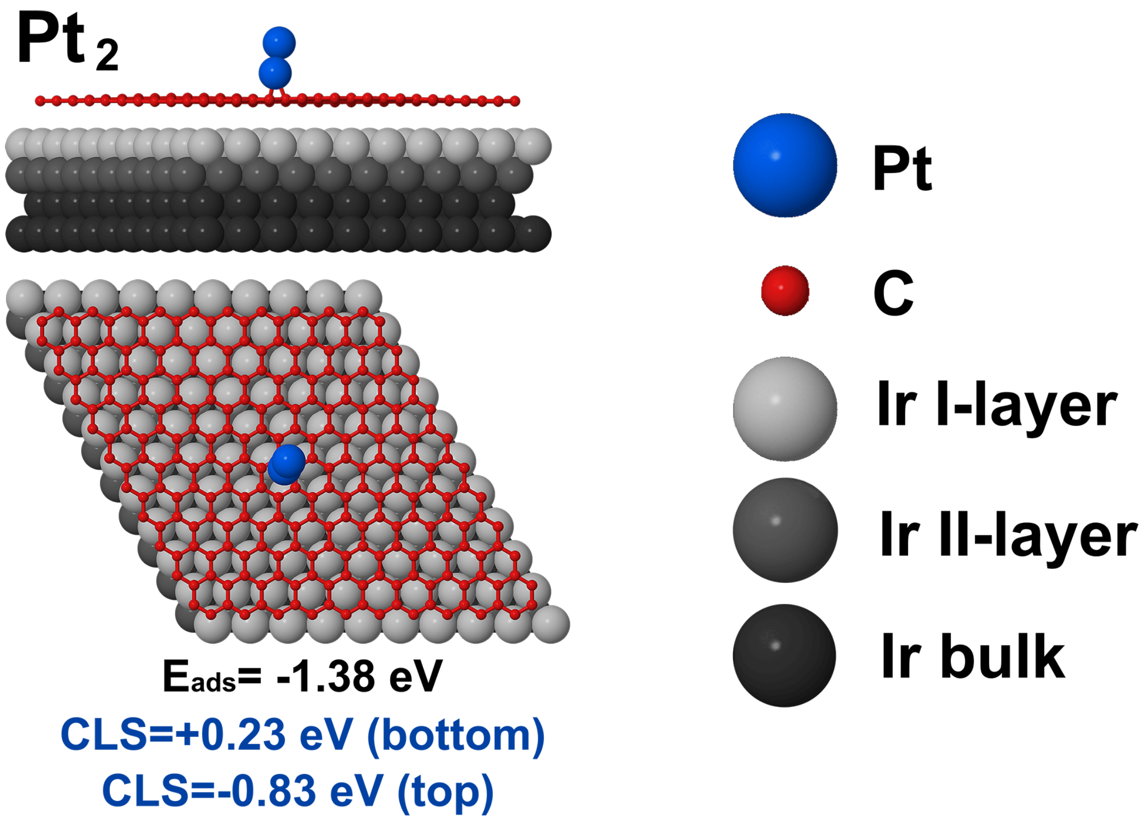

Figure S1: DFT calculated adsorption energies and Pt 4f CLSs for the lowest-energy Pt dimer (Pt_2) configuration adsorbed on Gr/Ir(111). The dimer adopts an upright geometry, with one Pt atom bonded to the graphene layer and the second positioned away from the surface. As reported in the legend Pt atoms are depicted in blue, C in red, while Ir atoms are showing a gradient from gray to black, depending on the layer.

Structural Models of Representative Graphene Defects

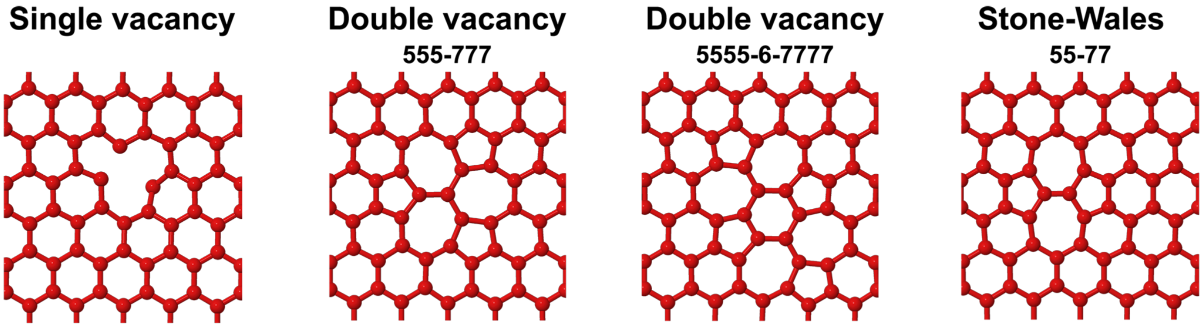

Figure S2: Structural models of the most representative defects in graphene considered in this study. Displayed are single vacancy (SV), two types of double vacancies (DVs), namely 555–777 and 5555–6–7777, and the Stone–Wales (SW) defect. Only carbon atoms (red) are shown for clarity. The underlying Ir substrate is omitted from the visualization but was fully included in the first-principles DFT calculations.

Adsorption Geometries of Pt Monomers at Graphene Defects

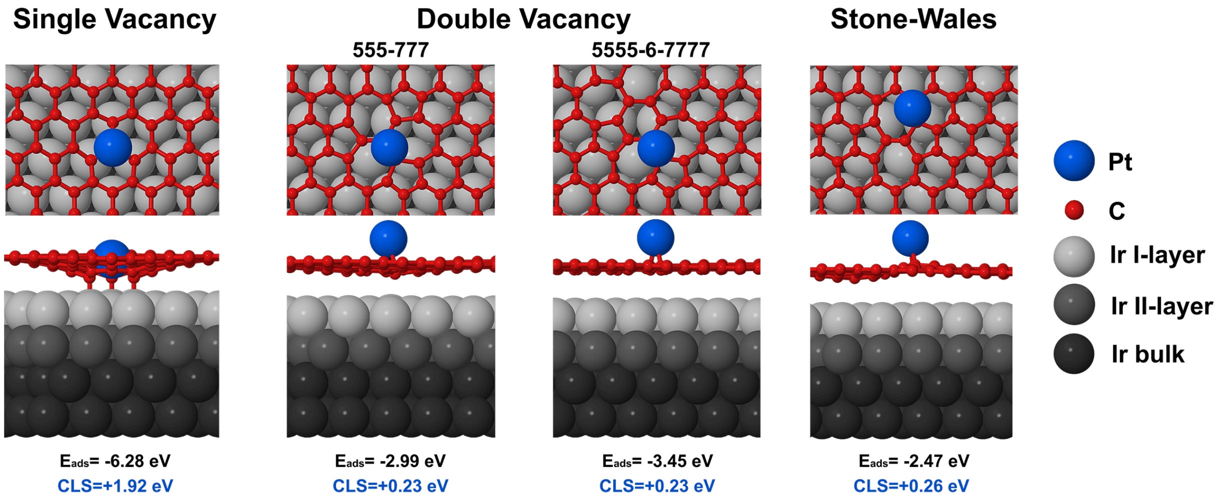

Figure S3: DFT calculated adsorption energies and $4f_{7/2}$ CLSs for Pt monomers adsorbed on different graphene defects, including single vacancy (SV), double vacancies (DV) of both 555–777 and 5555–6–7777 types, and Stone–Wales (SW) defects, reported in Figure S2. The Pt atoms adsorption sites range from quasi-hollow to bridge-like positions, with coordination to neighboring carbon atoms at the defect. As shown in the legend Pt atoms are depicted in blue, C in red, while Ir atoms are showing a gradient from gray to black, depending on the layer.

Additional Geometries of Pt atoms intercalated at Graphene Defects

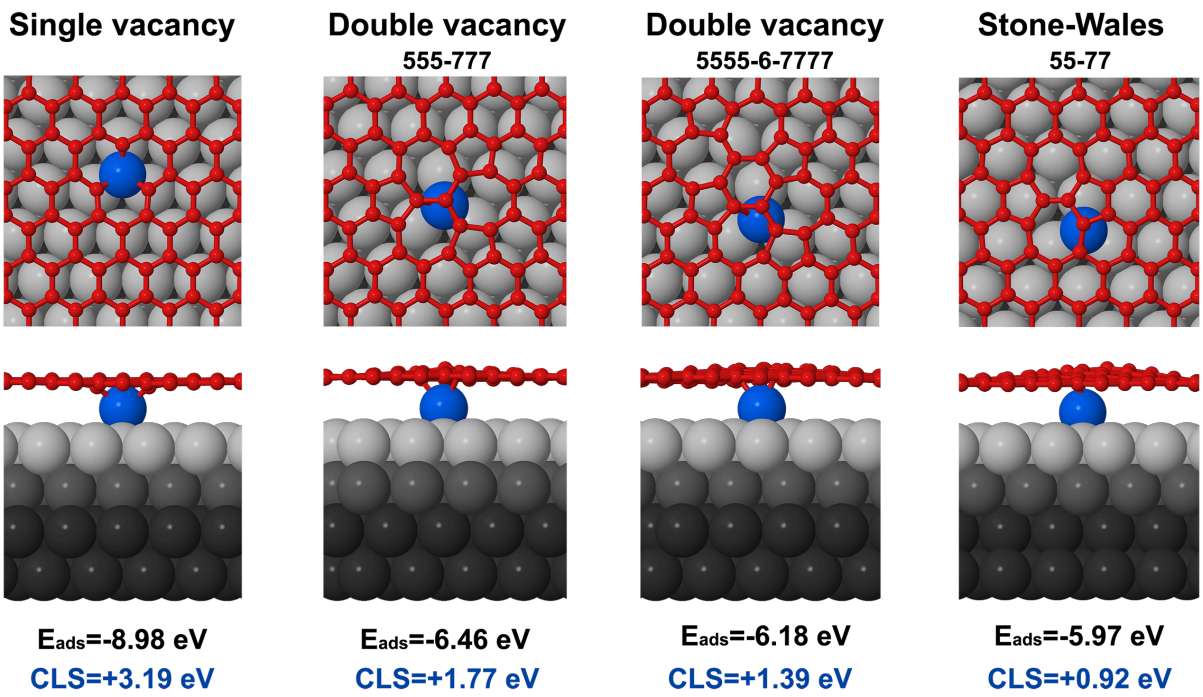

Figure S4: DFT-calculated adsorption energies and Pt $4f_{7/2}$ core-level shifts for Pt atoms sitting between the graphene defects and Ir substrate: single vacancy (SV), double vacancies (DVs) of the 555–777 and 5555–6–7777 types, and Stone–Wales (SW) defects. In the case of DVs and SW, one additional configuration per defect type is reported, extending the set shown in Figure 3 of the main manuscript. These additional geometries were selected to represent the extremal values in terms of adsorption energy and CLS among the configurations we investigated. Pt atoms are depicted in blue, C in red, while Ir atoms are showing a gradient from gray to black, depending on the layer.

Data Analysis of Pt $4f_{7/2}$ core level spectra: strategy for the determination of lineshape parameters

For an accurate and rigorous determination of the lineshape parameters of each Pt species contributing to the Pt $4f_{7/2}$ spectra, we applied a fitting strategy based on the analysis of the correlation matrix and χ^2 minimization. The method has been extensively and successfully applied for a variety of systems.[1,2] The binding energies (BEs) of all components were constrained to the DFT-predicted values. Specifically, the BE of the monomer component, used as the reference, was left completely free during the fit, defining the zero of the relative binding energy scale for all other components. The dimer contribution, corresponding to the planar Pt_2 configuration, was fixed at +0.15 eV, in agreement with the single CLS value predicted for this geometry. For larger clusters (Pt_n with $n \geq 3$), the BE was allowed to vary within the DFT-predicted range from +0.4 to +0.7 eV, reflecting the spread in CLS values due to the variety of local coordination environments. Similarly, for the intercalated Pt atoms, whose calculated CLS values fall between +0.90 and +1.39 eV depending on defect geometry, the BE was left free to vary within this interval. In both cases, the fitting was performed on the sum of the first eight spectra, where all Pt species are simultaneously present with sufficient signal-to-noise ratio. This yielded best-fit values of +0.65 eV for larger clusters and +1.10 eV for intercalated Pt, which were then adopted as fixed positions for the subsequent time-resolved analysis. Once the binding energies were determined, the relative intensities and lineshape parameters, such as Lorentzian width (Γ), asymmetry (α), and Gaussian width (G), were treated as free variables.

Although in principle a distinct lineshape could be expected for each Pt species, the limited signal-to-noise ratio due to the low coverages and needs of fast data acqusistion necessitated a reduction in the number of free parameters. To avoid overparameterization, we constrained monomers (M) and dimers (D) to share the same lineshape, and likewise adopted a common lineshape for larger clusters (L) and intercalated Pt (I). Linear background pa-

rameters (BG_1, BG_2) were also included in the fit.

To evaluate possible correlations among the different fitting parameters, we computed the correlation matrix shown in Figure S5. The matrix was calculated using the sum of the first 8 spectra to enhance the signal-to-noise ratio and because, in this range, all Pt species are present with non-negligible intensity.

For clarity, the regions of the matrix related to lineshape correlations are highlighted in different colors: the red square indicates internal correlations among monomer/dimer lineshape parameters ($\Gamma_{M/D}$, $\alpha_{M/D}$, $G_{M/D}$); the green square refers to those of larger clusters/intercalated Pt ($\Gamma_{L/I}$, $\alpha_{L/I}$, $G_{L/I}$); and the pink squares show cross-correlations between these two groups.

All correlation coefficients involving lineshape parameters were found to be well below 0.8, indicating weak interdependencies. As a result, we were able to proceed with one-dimensional χ^2 minimization maps for each parameter. Representative examples for the M/D species are shown in panel (b) of Figure S5, revealing the presence of well-defined minima for Γ, α, G. Similar behavior was observed for the L/I components.

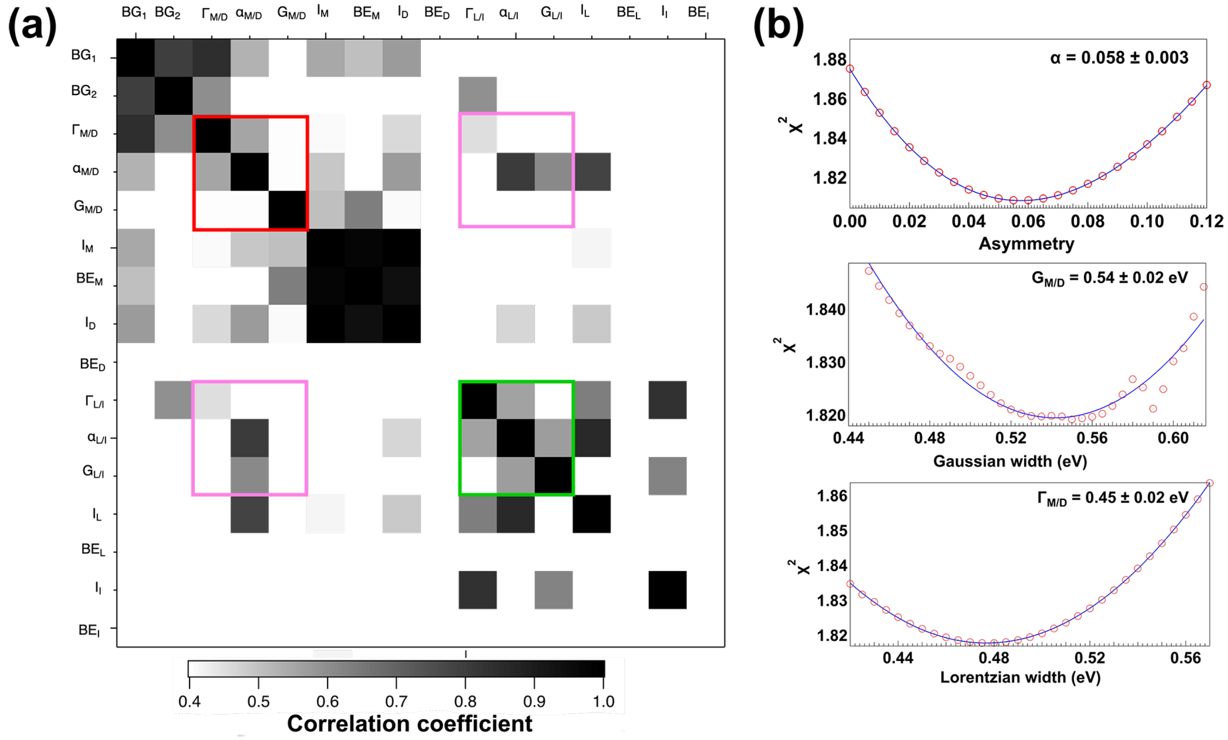

Figure S5: (a) Correlation matrix of the sum of the first 8 spectra. Labels refer to linear background (BG_1, BG_2), lineshape parameters (Γ, α, G) and intensities (I) for monomers (M), dimers (D), larger clusters (L) and intercalated Pt (I). Colored boxes highlight regions of the correlation matrix: red for M/D lineshape parameter correlations, green for L/I, and pink for cross-correlations. (b) One-dimensional χ^2 minimization curves for Γ, α, G of the M/D species.

Sensitivity of the kinetic model to the diffusion barrier E_d

To illustrate the sensitivity of the kinetic model to the diffusion barrier E_d, we simulated the monomer population by numerically solving the differential equations described in the manuscript using the experimental temperature (45 K). The diffusion barrier was varied in a range of ±20 meV around the best-fit value of 128 meV, in steps of 5 meV, as obtained for the higher-coverage dataset. Because the rate constants k_i follow an Arrhenius-like dependence ($k_i = \nu_0 \, e^{\left(-\frac{E_d}{k_B T}\right)}$), small variations in E_d directly translate into large changes in the predicted monomer decay. This occurs because k_1 (and, consequently, all k_i, since they are set as equal) sets the overall timescale of the process by controlling the jump frequency of the monomers. Figure S6 shows that even small deviations of ±10 meV from the best-fit barrier (128 meV) already result in substantial discrepancies with the experimental data, both in terms of timescale and the shape of the decay. This strong sensitivity further supports the robustness of the extracted diffusion barrier.

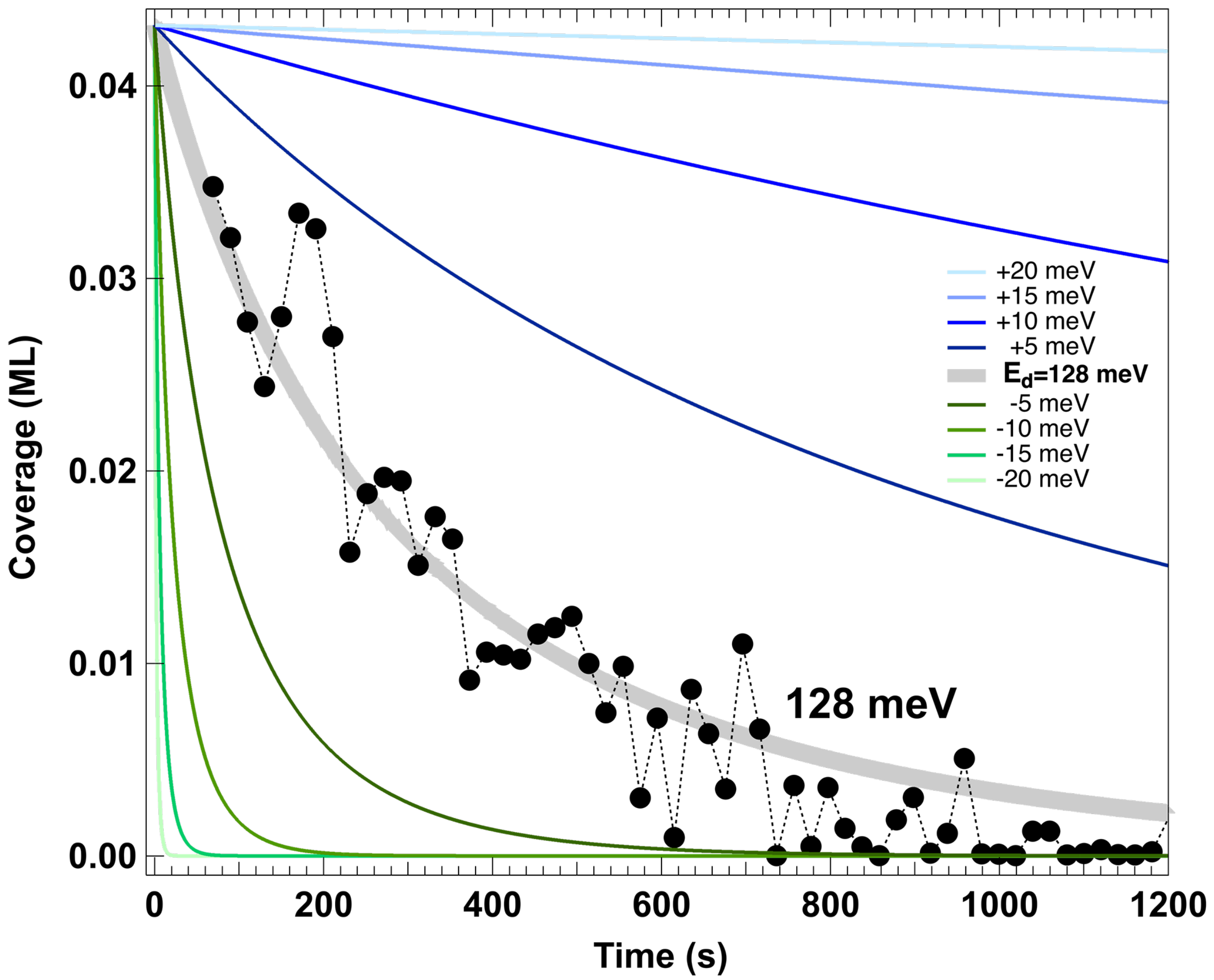

Figure S6: Experimental trend of the monomer population (black circles) compared with simulated decay curves obtained by numerically solving the kinetic model for different diffusion barriers E_d. The simulations were performed in 5 meV steps around the best-fit value of 128 meV: from the lightest to the darkest green, 20 meV (108 meV), 15 meV (113 meV), 10 meV (118 meV), and 5 meV (123 meV); the best-fit value is shown as a central gray line (128 meV); from the darkest to the lightest blue, +5 meV (133 meV), +10 meV (138 meV), +15 meV (143 meV), and +20 meV (148 meV). Even small deviations of ±10 meV result in noticeable discrepancies with the experimental data, both in terms of timescale and decay shape.

Experimental Results: low coverage data (0.04 ML)

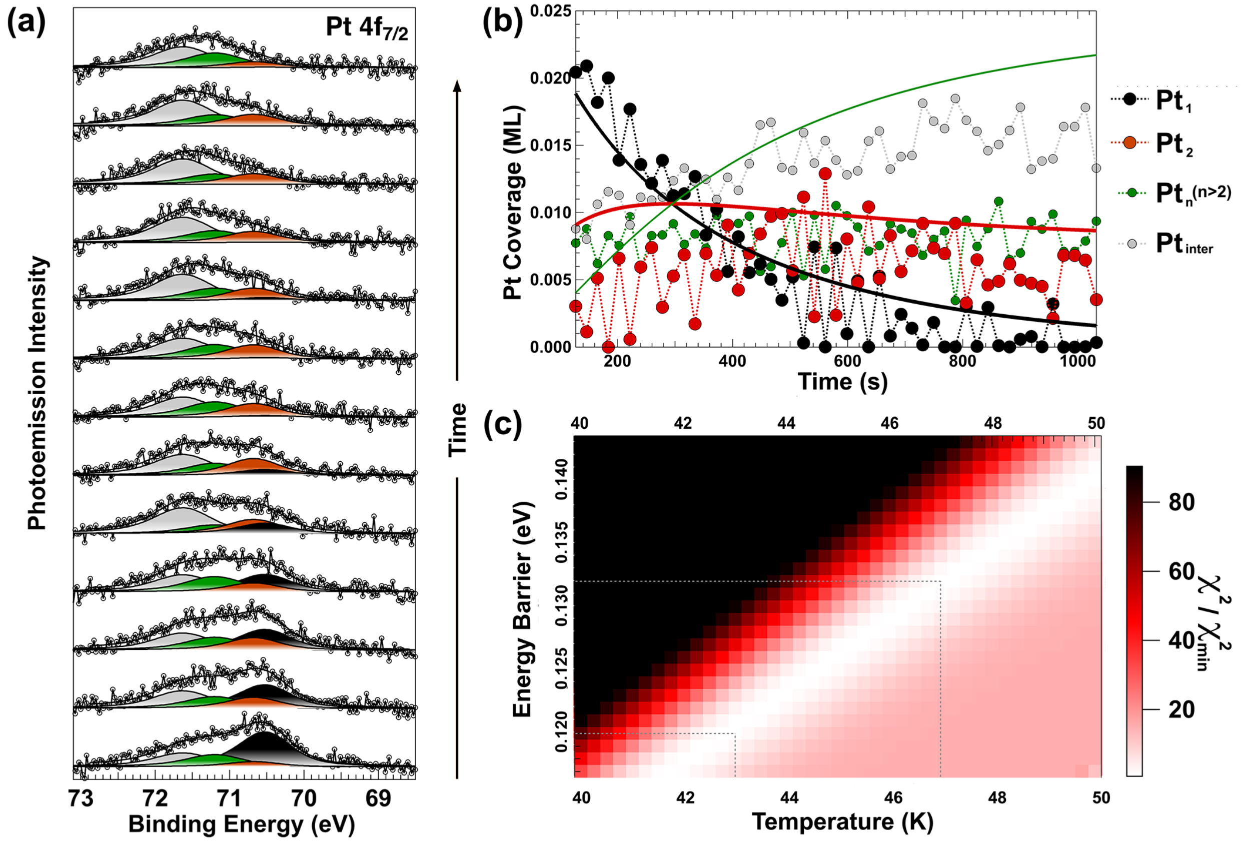

Figure S7: (a) Fit of the evolution of the $4f_{7/2}$ core-level spectra acquired in *real-time* with $h\nu = 200$ eV after Pt deposition at lower coverage (0.04 ML). For clarity, one out of every four spectra is shown, although the full dataset was used in the analysis. The deconvolution process allows identification of monomers (black), dimers (red), larger clusters (green), and intercalated Pt (gray). (b) Temporal evolution of the coverages of the different Pt species. Also shown is the best-fit solution from the kinetic model, corresponding to a temperature of 46 K and a diffusion barrier of 129 meV. (c) Two-dimensional χ^2 map illustrating the dependence of the fit on temperature T and diffusion barrier E_d, obtained by systematic variation of the parameters.

References

1. Ferrari, E.; Galli, L.; Miniussi, E.; Morri, M.; Panighel, M.; Ricci, M.; Lacovig, P.; Lizzit, S.; Baraldi, A. Layer-Dependent Debye Temperature and Thermal Expansion of

Ru(0001) by Means of High-Energy Resolution Core-Level Photoelectron Spectroscopy. *Phys. Rev. B* **2010**, *82*, 195420.

2. Baraldi, A.; Lizzit, S.; Novello, A.; Comelli, G.; Rosei, R. Second-Layer Surface Core-Level Shift on Rh(111). *Phys. Rev. B* **2003**, *67*, 205404.
